# Functional Differentiation of Floral Color and Scent in Gall Midge Pollination: A Study of a Schisandraceae Plant

**DOI:** 10.3390/plants11070974

**Published:** 2022-04-02

**Authors:** Shi-Rui Gan, Wei Du, Xiao-Fan Wang

**Affiliations:** College of Life Sciences, Wuhan University, Wuhan 430072, China; ganshirui@whu.edu.cn

**Keywords:** Cecidomyiidae, flower signal, gall midge, *Schisandra*, Schisandraceae

## Abstract

Gall midges are among the most host-specific insects. Their interactions with plants likely date back to the Cretaceous period. Plants from at least seven families are involved in gall midge pollination; however, little is known about the pollination signals of gall midges. In this study, we used a *Resseliella*–*Schisandra* model to investigate the roles of floral scent and color in attracting gall midges. Field observations, behavioral bioassays via Y-tubes, and “flight box” experiments were performed. The results demonstrated that gall midges may be attracted by both floral scent and color and that two flower signals are more effective in promoting insect flower-landing than either alone. In the field, gall midges visited male flowers effectively at night but almost always visited female flowers during the day. Thus, during the *Resseliella*–*Schisandra* interactions, female flowers predominantly employed visual cues over scent to attract midges during the day; in contrast, olfactory cues were more functional for male flowers to export pollen in the dark. In this study, we first identified the roles of floral color and the functional differentiation of visual and olfactory cues during gall midge pollination.

## 1. Introduction

Cecidomyiidae (Diptera), commonly known as gall midges, originated from the Jurassic Period and comprise 6651 known species worldwide, approximately three-quarters of which are phytophagous [1,2]. The interaction between Cecidomyiidae and plants originated early in the Cretaceous era and has expanded greatly with respect to flowering plants in the Tertiary era [2,3]. Some Cecidomyiidae species have formed a stable relationship with their host plants during the co-evolution process, for example via galls induced by the larvae of gall midges [2,4,5] or through pollination (Table 1) [6]. 

Effective interactions between plants and pollinators facilitate the sexual reproduction of angiosperms. Floral trait diversity (color, scent, etc.) is often believed to be the basis of selection for pollinators [7,8]. Plants employ floral color [9,10], floral scent [11,12,13], or floral shape [14,15,16] to attract pollinators. Thus, studies on pollination signals are very important for understanding plant–pollinator interactions. To date, midge pollinators have been found in 37 species of the seven families, 27 of which belong to Schisandraceae (Table 1) [6]. Midge pollinators visit flowers for nectar [17,18], pollen [19,20], and liquid secreted by glandular trichomes [21,22] or to lay eggs in flowers [6,23,24,25,26]. However, we still know little about the pollination signals for gall midges.

Numerous studies have attempted to address the roles of floral scent or color in attracting gall midge pollination. For example, *Kadsura longipedunculata* flowers attract midges mainly by relying on floral odor because flower visiting occurs mostly at night, although color acts as an attractant in the daytime [27]. Conversely, the visual cues of *Schisandra henryi* are thought to play a major role in attracting pollinators owing to the odorless characteristics of flowers [28]. In Moraceae, the floral odor of *Artocarpus integer* was deemed to be an attractant of midges because both male and female inflorescences produce a fruit-like unpleasant smell and female inflorescences provide no rewards for gall midges [29]. Recently, two studies confirmed the role of floral odor in attracting gall midges via Y-tubes in *Artocarpus heterophyllus* [26] and *Kadsura. coccinea* [6]. However, the information on pollination signals for gall midges obtained from most studies is only speculative and lacks study data. Furthermore, none of these cases has analyzed the role of flower color in gall midges via bioassays. 

**Table 1:** Plant species pollinated by gall midges (Cecidomyiidae): *S* = *Schisandra*; *K* = *Kadsura*; *I* = *Illicium*.

Plant Species	Plant Family	Gall Midges	Pollination Rewards	Flower Odor and Time	Visiting Time	References
*S. henryi*	Schisandraceae	*Resseliella* sp.	Pollen	No odor (by human smell and GC-MS)	Daytime	[19,28]
*S. sphenanthera*		*Resseliella* sp.	Brood site	Sweet odor day and night	Day and night for male flowers; day for female flowers	[30]
*S. repanda*		*Resseliella* sp.	Pollen	Sweet odor day and night	04:00–12:00 for male flowers; 07:00–12:00 for female flowers	[19]
*S. bicolor*		*Resseliella* sp.	Pollen	Odor from 19:00–22:00, but no odor after 06:00	18:00–23:00	[19]
*K. longipedunculata*		*Megommata* sp.	Pollen	Odor from 21:00–02:00	21:00–02:00	[27]
*K. coccinea*		*Resseliella* sp.	Brood site	Strong odor during the first two nights	19:00–04:00	[25]
*K. heteroclita*		*Resseliella* sp.	Brood site	Strong odor during the first two nights	22:30–04:00	[25]
*K. oblongifolia*		*Resseliella* sp.	Brood site	Strong odor during the first two nights	20:00–04:00	[25]
*I. floridanum*		*Micromya* sp.; *Clinodiplosis* sp.	Nectar, possibly pollen	Odor like freshly caught fish or wine	All day, more at dusk	[17]
*I. parviflorum*		*Clinodiplosis* sp.; *Giardomyia* sp.; *Lestodiplosis* sp.	Nectar, possibly pollen	Faint sweet odor	All day, more at dusk	[18]
*I. henryi*		*Resseliella* sp.	Brood site, fungus mycelia	No odor detectable by human smell	All day, more at dusk	Du Unpub. data
*I. dunnianum*; *I. tsangii*		*Clinodiplosis* sp.	Brood site (flower heating)	No odor detectable by human smell	Mostly at night	[31]
*I. lanceolatum; I. majus; I. oligandrum; I. petelotii; I. verum*		*Resseliella* sp.	Brood site	Unknown	Unknown	[6]
*Amborella trichopoda*	Amborellaceae	*Asphondylia* sp.	Brood site (female flowers), pollen	Scent like licorice or feces	At night	[24]
*Siparuna* sp.	Monimiaceae	*Asynapta* sp.; *Clinodiplosis* sp.; *Dasineura* sp.	Brood site (on male flowers)	Strong lemon scent	At night	[32,33,34]
*Piper novae-hollandiae*	Piperaceae	*Contarinia* sp.	Brood site (on male inflorescence)	Sweet scent day and night	At night	[23]
*Aspidistra xuansonensis*	Asparagaceae	Species of cecidomyiid	Brood site, pollen	No odor by human smell	9:00–14:20	[20]
*Artocarpus heterophyllus*	Moraceae	*Clinodiplosis ultracrepidata*	Brood site (on male inflorescence), fungus mycelia	Primarily methyl 2-methylbutyrate, methyl isovalerate, and methyl tiglate	Unknown	[26]
*Artocarpus integer*		*Contarinia* sp.	Brood site (on male inflorescence), fungus mycelia	Odor similar to ripe watermelon scent between 18:00–20:00	Mostly at night	[29]
*Theobroma cacao*	Sterculiaceae	*Clinodiplosis* sp.; *Mycodiplosis* sp.	Liquid secreted by glandular trichomes on the ovary	No odor detectable via human smell	At night and early morning	[21,22]

Gall midges and Schisandraceae have been interacting since at least the Early Miocene era based on molecular clocks [6]. Schisandraceae plants predominantly exhibit a species-specific relationship with gall midge pollinators, especially with species of the genus *Resseliella* (Table 1) [6,25]. In this study, we used *Schisandra sphenanthera* and its obligate pollinator *Resseliella* sp. as a model system to study the relationships between floral stimuli and gall midge behaviors. A study conducted by Luo et al. [6] proved, via sequencing of the COI arthropod barcoding marker, that larvae and adult midges in *S. sphenanthera* flowers, which were collected at three locations in three provinces in China, are one species, which indicates that there is a strictly specific-species relationship between *S. phenanthera* and *Resseliella* sp. There were 17.6 ± 5.3 and 17 ± 4.9 midge eggs in male and female flowers of *S. sphenanthera*, and the “brood site” was the pollination reward for gall midges [6]. We attempted to determine if gall midges are attracted by floral scent and color, and if so, we tried to ascertain the nature of the relationship between visual and olfactory cues in gall midge pollination. We also wanted to discover whether floral scent and color have the same effect for male and female flowers in attracting gall midges in natural conditions. 

## 2. Results

### 2.1. Gall Midge Reaction to Floral Scent and Color

In the Y-tube olfactometer tests, at least 52.2% of midges chose between the two arms, all of which exhibited significantly more attraction to the floral scent than the air control; (Ym (yellow male flower), *χ2* = 28.57, df = 1, *P* < 0.001; Yf (yellow female flower), *χ2* = 7.69, df = 1, *P* = 0.006; Rm (red male flower), *χ2* = 16.10, df = 1, *P* < 0.001). In the box experiments, gall midges did not exhibit a preference between Ym and Rm flowers under dark conditions (*Z* = −0.43; n = 20; *P* = 0.67) but presented a significant preference for Rm flowers under light conditions in group 1 (*t* = 5.20; df =18; *P* < 0.001), suggesting that midges can distinguish between red and yellow colors and demonstrate a higher preference for the red color (Table 2). In group 2, under light or dark conditions, Ym flowers were all more attractive to midges than Yf flowers (light, *Z* = −2.84; n = 20; *P* = 0.004; dark, *Z* = −3.20; n = 20; *P* = 0.01). However, this preference towards male flowers was lost when flowers were placed in glass vessels (*t* = 0.13, df = 18, *P* = 0.90), indicating that the olfactory cues of male flowers differ from those of female flowers. Additionally, both olfactory cues (in the dark) or visual cues (in the glass vessels), when acting in isolation, could only trigger minimal midge flower-landing (13.12–22.94%), whereas both cues, when offered together, resulted in proportionally more frequent flower-landing (67.70−71.39%).

### 2.2. Visiting Preference for Male and Female Flowers in the Natural Community

**Figure 1:**
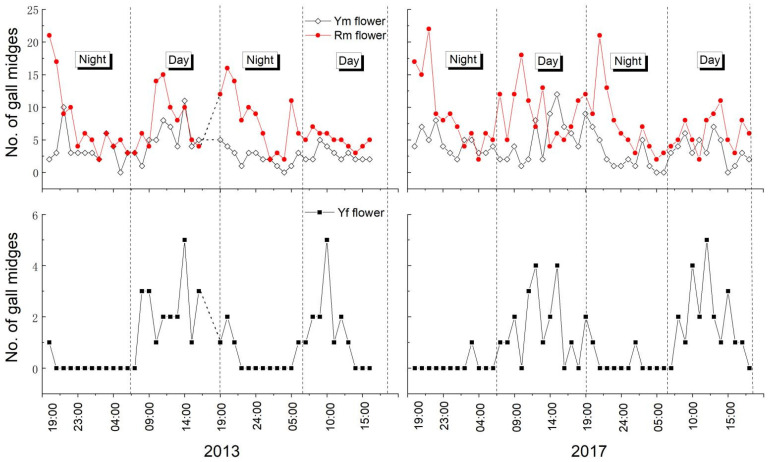
Visiting dynamics of gall midges on the three flower phenotypes of *Schisandra sphenanthera* under natural conditions.

In the field, more midges were observed on male flowers than on female flowers during the day and the night, and Rm flowers were more attractive to midges than both Ym and Yf flowers (Figure 1). Generally, there was an average of 0.96 ± 0.20 (n = 44) midges in each female flower, but 3.41 ± 0.35 and 7.27 ± 0.67 (n = 44) midges were found in each yellow and red male flower, respectively (based on data from 2013). Few midges were found in female flowers at night, and the visits to female flowers exhibited a “day–night movement rhythm”, indicating that color might be more functional for female flowers. This phenomenon has been observed in at least two of the study years (Figure 1).

## 3. Discussion

### 3.1. Floral Odor in Gall Midge Pollination

Diverse sensilla types of gall midges (sensilla coeloconica, sensilla trichodea, and sensilla circumfila) are thought to have an olfactory function [35]. In non-pollination interactions, female *Resseliella theobaldi* exhibit a strong preference for splits in raspberry canes [36,37] and *Orseolia oryzivora* are attracted by odors emitted from intact rice plants [38]. For plants pollinated by midges, at least 60% of species have floral scents (Table 1). Luo et al. [25] and Gardner et al. [26] have confirmed the role of floral odor in the responses of gall midges to *A. heterophyllus* and *K. coccinea*, respectively, via a two-choice glass Y-tube olfactometer. *S. sphenanthera* flowers produce scents during the life cycle [30]. Our results via Y-tube tests were consistent with those of Luo et al. [25] and Gardner et al. [26]. Gall midges were attracted by the flower scent, and there was a visiting peak for male flowers after flowering at dark (Figure 1). Though gall midges can still be found in male flowers in the daytime, most pollen was gone the next morning, which means that the pollination efficiency of insects with respect to male flowers during daytime was very low. Combined with the results of the box experiments (Ym and Yf under light and dark fields), we deduced that olfactory cues might be more crucial for pollen exportation in male flowers than floral color (Table 2, Figure 1). The gall midges found in male flowers in the daytime could rest or lay eggs [6]. 

In general, the life span of gall midge adults is very short (generally 1−2 days). Adult gall midges must finish copulation and oviposition, and then find the right host plants soon after. These features require efficient mechanisms for insects to find mates or hosts, such as using olfactory cues [38,39,40,41]. The different visiting preferences between male and female *S. sphenanthera* flowers imply the difference in floral odor among them (Table 2). According to the current studies of gall midge pollination, the main volatile components of flowers appear to be diverse [22,25,27,42]. For instance, methyl butyrate is the dominant compound for *K. longipedunculata* flowers [27]. but α-pinene is the compound for *K. coccinea* [25]; methyl 2-methylbutyrate, methyl isovalerate, and methyl tiglate are compounds for *Artocarpus heterophyllus* [26]; and tridecane, pentadecane, (Z)-7-pentadecene, and (Z)-8-heptadecene are compounds for *Theobroma cacao* [42].

Although floral fragrance emission is very common in gall midge-pollinated plants, odor emission rhythm and insect visiting times differ among species (Table 1). For example, *S. bicolor* and *K. longipedunculata* emitted floral scents and were pollinated only at night [19,27]. Gall midges visit *Illicium floridanum* and *I. parviflorum* flowers during both day and night [17,18]. Moreover, for some plants, such as *S. sphenanthera* and *S. repanda*, floral scents are produced throughout the lifespan of flowers, whereas gall midge visits to male and female flowers occur during different time periods (Figure 1) [19]. Gall midges are considered to be among the most host-specific insects (Table 1) [43]. This characteristic exists between most studied Schisandraceae species and gall midges, which was confirmed by sequencing the COI arthropod barcoding marker in larvae and adult midges found in Schisandraceae flowers [6]. In the wild, many Schisandraceae species, including *S. sphenanthera*, grow within meters of each other and have overlapping flowering times [6]. The different floral scent components and emission rhythms for different species could be an adaption to the obligate gall midge pollination mutualism and a mechanism to maintain species stability.

### 3.2. Floral Color in Gall Midge Pollination

Odorless plants that are visited by gall midges during the daytime might indicate the role of visual stimuli in flower visitation (e.g., *Aspidistra xuansonensis* and *S. henryi*) [20,28]. Some authors have speculated that *Theobroma cacao* flowers, which have *Clinodiplosis* sp. and *Mycodiplosis* sp. (Cecidomyiinae), as potential pollinators, might attract pollinating insects through brightly colored petal ligules and distinctive ultraviolet light-absorbing/reflecting properties [21,22,44]. In this study, the dark context could lower the landing rate of gall midges in the box experiments, suggesting that visual cues might facilitate insects landing on flowers. For female flowers, insect visiting in the field almost always occurred during the daytime (Figure 1). When compared with floral scents, visual cues seemed to be more functional for female flowers.

To date, plants with gall midges as pollinators have been found in the ANA grade (Amborellaceae and Schisandraceae (including Illiciaceae)), magnoliids (Piperaceae, Monimiaceae), monocots (Asparagaceae), as well as core eudicots (Moraceae and Malvaceae (incl. Sterculiaceae)). However, they are more prevalent in the ANA grade (Table 1). These flowers vary greatly in color, from single cream (e.g., Amborellaceae) to the broadest range of perianth pigmentation (e.g., Malvaceae). These plants usually attract insects using floral scent and colored tepals [45]. However, a few studies were referred to in the relationship between midges and floral colors. A few insects found in *Kadsura longipedunculata* may be attracted to it by virtue of its color in daylight hours, but the attraction of most gall midges (*Megommata* sp.) was deemed to correlate with the nocturnal production of heat and fragrance [25]. In *Schisandra henryi*, while pollen is the reward for midges in male flowers, female flowers are thought to attract pollinator insects using visual cues to deceive them, since they offer no food [26]. However, Luo et al. [25] denied all speculations that pollen is the pollination reward for gall midges, including in the cases of *S. henryi* and *S. sphenanthera* [6]. In Schisandraceae, the flowers of some species have a scent while others have no smell. Moreover, gall midges are observed as having different visiting rhythms for different plant species (Table 1). Thus, Schisandraceae plants provide an ideal phytogroup to study the relationship between flowers and Cecidomyiidae pollinators.

## 4. Materials and Methods

### 4.1. Species Studied

**Figure 2:**
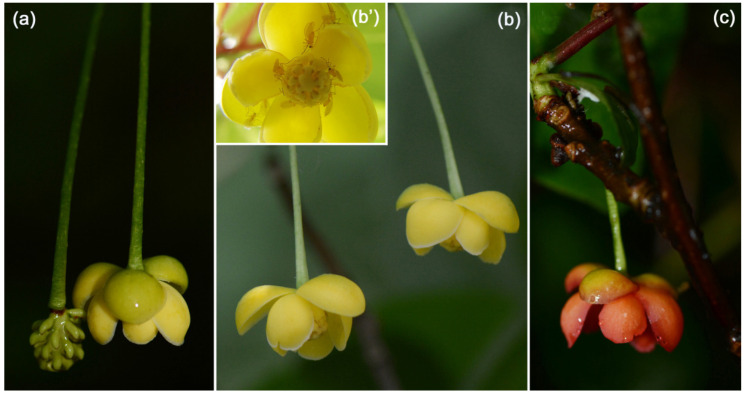
Phenotypes of *Schisandra sphenanthera* flowers: (**a**) yellow female flower; (**b**) yellow male flower and *Resseliella* pollinators (**b’**); (**c**) red male flower.

*Schisandra sphenanthera* Rehd. & Wils., a perennial, dioecious, woody liana found in central China, flowers from early May for approximately 3 weeks and is obligately pollinated by *Resseliella* sp. [30]. The most common flower color is yellow (present in both female and male flowers; Figure 2a,b), but flowers of some male individuals are red (only 5.92% of all individuals investigated) (Figure 2c) [30]. Male and female flowers open almost simultaneously at approximately 19:00 and release an obvious fragrance during the day as well as in the night. Anthers dehisce flowers open 0.5–1 h after, and pollen can be easily shed by gently tapping the flowers or when insects move on the androecia [30]. The stamens were almost empty of pollen the next morning. This study was conducted on Jigong Mountain (Henan Province, China) in 2013 and 2017. Herbarium vouchers of *S. sphenanthera* (No. 08114) were deposited at the Herbarium of Wuhan University (WH). *Resseliella* belongs to the subfamily Cecidomyiinae and comprises 56 species worldwide [2,46]. At present, at least 27 plants in three genera (*Schisandra*, *Kadsura*, and *Illicium*) of Schisandraceae are pollinated by *Resseliella* spp., and most species have their own midge pollinator (Table 1) [6,25].

### 4.2. Y-Tube Olfactometer Tests

To test if midges are attracted by floral scents, Y-tube olfactometer experiments were conducted in the dark and at room temperature. The Y-tube equipment was set up as described by Bertschy et al. and Tooker et al. [47,48], wherein the Y-tube was made of quartz glass tubing (1.5 cm internal diameter) and consisted of a main tube (20 cm long) and two selecting arms (15 cm long) at an angle of 75°. Compressed air was pushed by an atmospheric sampler and was passed through an activated charcoal filter and distilled water before entering the two glass cylinders containing the odor source. The air current was maintained at 100 mL min^−1^ by two flowmeters.

Midges were collected in the wild. Before testing, the insects were placed in the dark for 30 min. Each midge was individually placed in the entry of the horizontally oriented Y-tube and was given 5 min to choose. When the individual had moved into one arm to a distance 2 cm from the Y-junction point, the choice was recorded. If the midge remained in the main arm or had not reached the decision point in the given time, it was recorded as “no choice”. Fresh flowers versus air control were used as the odor source. Each midge was used only once, and 40 insects were used for each phenotype (Ym, Rm, and Yf). To neutralize any optical or asymmetrical effects of the olfactometer on insect behavior, both arms and odor chambers were switched every five assays to eliminate directional bias. Before the next bioassay, the Y-tube olfactometer and odor chambers were rinsed with 95% ethanol and distilled water and then dried with a hairdryer.

### 4.3. Flight Boxes and Behavioral Bioassays

**Figure 3:**
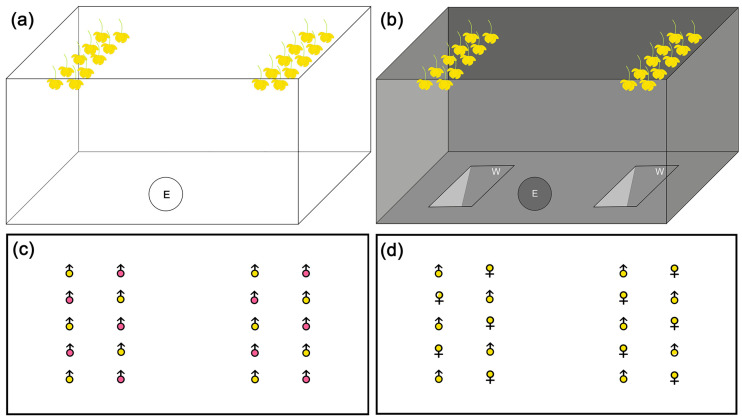
“Flight box” biotests. (**a**) Light-field box made of transparent polyethylene terephthalate (TPT). Fresh flowers hung on the roof plate via two lines of holes (line space = 4 cm; hole space = 2 cm; 3.5 and 5 cm from the two edges) (**b**) Dark-field boxes (TPT box placed inside a non-transparent (NT) box made of corrugated paper). E = insect entrance on the lateral side of boxes, 3 cm diameter; W = windows at the bottom plate of the NT box, which were used for insect landing observations under a lamplight. Windows were closed during testing. (**c**,**d**) Flower arrangement patterns on the roof plate as follows: (**c**) red and yellow male flowers; (**d**) yellow male and female flowers.

To evaluate the behavioral responses of midges to visual and olfactory cues and to determine the relationship of the two floral stimuli under light and dark conditions, dual-choice bioassays were conducted in two self-made “flight boxes” during 14–16 May, 2017. One was made with transparent polyethylene terephthalate (TPT box; 30 cm × 15 cm × 15 cm), and the other was a non-transparent box made with corrugated paper (NT box; 30 cm × 15.5 cm × 15.5 cm). In the transparent TPT box, midges could detect both visual and olfactory cues (Figure 3a). To simulate dark conditions, the TPT box was placed into the NT box (Figure 3b), where midges could only sense olfactory cues. Behavioral bioassays were conducted for the following treatments: Group 1, with Ym and Rm flowers under light and dark conditions; Group 2, with Ym and Yf flowers under light and dark conditions and with Ym and Yf flowers in glass vessels (visual cues only) under light conditions.

Twenty fresh flowers (with copious pollen for male flowers and bright yellow petals for female flowers) of similar sizes were selected and hung on the box, as in Figure 3. Pedicles were bagged with slightly wet cotton to delay the wilting of flowers. Each group of flowers was used for only one treatment (e.g., “Ym and Rm flowers under light conditions” as a “treatment”). In each bioassay, 19–57 midges captured within 30 min each time were used and 10 groups of insects were used for each treatment. The number of midges landing on the flowers was recorded 2 min later when insects flew into the box from the entrance (E) on the lateral side of the boxes (Figure 3a,b) and stayed in flowers.

### 4.4. Flower Visitor Field Observations

To determine the visiting preferences of gall midges on flowers, 10 flowers of each phenotype (Yf, Ym, and Rm) were observed randomly in the field at hourly intervals during both day and night on 13 and 14 May 2013, and on 15 and 16 May 2017. The number of gall midges in each flower was recorded. A flashlight covered with thin red plastic film was used for nocturnal observations.

### 4.5. Statistical Analyses

We used the Chi-squared test to compare the attraction of different flower phenotypes to the air control in the Y-tube olfactometer tests. Independent samples *t*-tests, with assumed equal variances, were used to analyze the behavior of gall midges in the flight boxes. If the data in the box experiments were not normally distributed, a Mann–Whitney U test was used instead. All statistical analyses were conducted using the IBM SPSS 19.0 statistical software package (IBM, Armonk, NY, USA).

## 5. Conclusion and Prospects

Plants from at least seven families are involved in gall midge pollination; however, little is known about the pollination signals for them. In the present study, we first identified the roles of floral color and the functional differentiation of visual and olfactory cues to gall midges during the *Resseliella–Schisandra* interaction. Our results showed that gall midges could be attracted by both floral scent and color. Elimination of either floral color (via a dark field) or scent (via glass vessels) may lower the flower-landing rates of gall midges. Moreover, under natural conditions, gall midges showed a “day–night movement rhythm to female flowers” pollination mode, indicating that the visual cues were predominantly employed by female flowers over scent to attract midges during the day. In contrast, olfactory cues were more functional for male flowers to export pollen in the dark based on the male flower phenology and insects visiting activity.

Though have identified the role of floral color and scent in pollination, certain aspects are still unknown, such as why so many gall midges visited male flowers over female ones, even though both flowers serve midges equally well for “brooding” ( 17.6 ± 5.3 and 17 ± 4.9 midge eggs were found in male and female flowers of *S. sphenanthera*) [6] and why gall midges showed a day–night movement rhythm for female flower visitation. Further research may be necessary to detect the relationship between gall midges and flowers by GC-EAD (gas chromatography-electroantennographic detection). Moreover, additional field studies are needed to determine whether the “day–night movement rhythm to female flowers” pollination mode occurs in other species of Schisandraceae. A more complete understanding of the evolution of floral signals in response to pollinators of basal angiosperm should shed light on evolutionary processes that mediate the massive radiation of angiosperms.

## Figures and Tables

**Table 2:** Visiting response (mean ± SE) of gall midges to *Schisandra sphenanthera* flowers in the box experiments. The difference ^†^ among flowers was assessed by the independent sample *t*-test; non-normal distributions were tested using the Mann–Whitney U test (Ym = yellow male, Rm = red male, Yf = yellow female).

Group	Flower	Light Field (n = 10 )	*P*	Dark Field (n = 10 )	*P*	Light Field with Flowers in Glass Vessels (n = 10 )	*P*
Group 1	Ym flower	6.20 ± 1.36	<0.001 ^†^	3.20 ± 0.44	0.67	-	-
	Rm flower	15.60 ± 2.22		2.90 ± 0.43		-	
Group 2	Ym flower	15.50 ± 2.38	0.004	2.70 ± 0.56	0.001	2.70 ± 0.54	0.90 ^†^
	Yf flower	7.70 ± 1.28		0.50 ± 0.22		2.60 ± 0.52	

## Data Availability

The data presented in this study are openly available in Mendeley Data at DOI: 10.17632/d4kfkw96sk.1.
